# Development of a simplified RT-PCR without RNA isolation for rapid detection of RNA viruses in a single small brown planthopper (*Laodelphax striatellus* Fallén)

**DOI:** 10.1186/s12985-017-0732-6

**Published:** 2017-05-03

**Authors:** Qiufang Xu, Haoqiu Liu, Pingping Yuan, Xiaoxia Zhang, Qingqing Chen, Xuanli Jiang, Yijun Zhou

**Affiliations:** 1 Institute of Plant Protection; Jiangsu Academy of Agricultural Sciences; Jiangsu Technical Service Center of Diagnosis and Detection for Plant Virus Diseases, Nanjing, Jiangsu People’s Republic of China; 2 College of Agriculture, Guizhou University, Guiyang, Guizhou People’s Republic of China

**Keywords:** Small brown planthopper, Virus detection, Simplified RT-PCR

## Abstract

**Background:**

The small brown planthopper (SBPH) is an important pest of cereal crops and acts as a transmission vector for multiple RNA viruses. Rapid diagnosis of virus in the vector is crucial for efficient forecast and control of viral disease. Reverse transcription polymerase chain reaction (RT-PCR) is a rapid, sensitive and reliable method for virus detection. The traditional RT-PCR contains a RNA isolation step and is widely used for virus detection in insect. However, using the traditional RT-PCR for detecting RNA virus in individual SBPHs becomes challenging because of the expensive reagents and laborious procedure associated with RNA isolation when processing a large number of samples.

**Results:**

We established a simplified RT-PCR method without RNA isolation for RNA virus detection in a single SBPH. This method is achieved by grinding a single SBPH in sterile water and using the crude extract directly as the template for RT-PCR. The crude extract containing the virus RNA can be prepared in approximately two minutes. Rice stripe virus (RSV), rice black streaked dwarf virus (RBSDV) and Himetobi P virus (HiPV) were successfully detected using this simplified method. The detection results were validated by sequencing and dot immunobinding assay, indicating that this simplified method is reliable for detecting different viruses in insects. The evaluation of the sensitivity of this method showed that both RSV and HiPV can be detected when the cDNA from the crude extract was diluted up to 10^3^ fold. Compared to the traditional RT-PCR with RNA isolation, the simplified RT-PCR method greatly reduces the sample processing time, decreases the detection cost, and improves the efficiency by avoiding RNA isolation.

**Conclusions:**

A simplified RT-PCR method is developed for rapid detection of RNA virus in a single SBPH without the laborious RNA isolation step. It offers a convenient alternative to the traditional RT-PCR method.

**Electronic supplementary material:**

The online version of this article (doi:10.1186/s12985-017-0732-6) contains supplementary material, which is available to authorized users.

## Background

The small brown planthopper (SBPH), *Laodelphax striatellus* Fallén (Delphacidae: Hemiptera), is a serious sap-sucking pest of agricultural crops. More importantly, it acts as an insect vector to transmit multiple plant viruses and causes severe yield losses. For example, it transmits reoviruses (RBSDV and maize rough dwarf virus) [1, 2], tenuiviruses (rice stripe virus, RSV) [3], rhabdoviruses (barley yellow striate mosaic virus and northern cereal mosaic virus) [4, 5], and cripaviruses (Himetobi P virus, HiPV) [6]. Both RSV and RBSDV are transmitted by SBPH in a persistent, circulative and propagative manner. RSV can be transmitted by SBPH from mother to offspring [7], while RBSDV cannot. The rice stripe disease caused by RSV damaged over 957,000 hectares of paddy fields in 2003 and 1,571,000 hectares in 2004, accounting for 80% of the rice fields and a 30–40% yield loss in China [8]. RBSDV not only infects rice plants to cause rice black-streaked dwarf disease, but also causes maize rough dwarf disease in maize [1]. These viral diseases have been economically destructive in the rice- and maize- growing areas in China for decades [9].

Rapid diagnosis of virus in vector is important for viral disease forecast and control. Various approaches have been developed for detection of RNA virus in its insect vector, including biological inoculation [10, 11], direct observation using electron microscopes [3, 12], antibody-based serological method [13], and other molecular detection methods. Biological inoculation method is time consuming and labor-intensive; for example, it takes approximately one month for the plants to show disease symptoms after inoculation with RBSDV via viruliferous insects [11]. Electron microscopes are very expensive and require specialty-trained personnel to operate them. In addition, the results of electron microscopy usually need to be confirmed by other methods [12]. Serological methods, such as enzyme-linked immunosorbent assay, are economical for detection of high throughput samples [14], but they are limited by the specificity and availability of antibodies against the virus. Deep sequencing and qRT-PCR and are of high sensitivity and specificity [15–17], but expensive. RT-PCR is a rapid, specific and reliable assay to detect RNA viruses [18, 19], especially for viruses that do not have antibodies available.

Traditional RT-PCR assays usually require purified RNA for reverse transcription. Isolation of RNA with commercial kits is expensive and time consuming. Besides, as SBPH is a small insect, measuring approximately 2–4 mm long, it is challenging to purify RNA from an individual SBPH. In this study, we developed a simplified RT-PCR assay for RNA virus detection in a single SBPH without RNA isolation. The sensitivity and reliability of this detection method are assessed and compared with those of traditional RT-PCR.

## Methods

### Preparation of SBPH used for virus detection

SBPHs free of RSV and RBSDV has been continuously maintained in our lab over ten years. A RSV-viruliferous SBPH population, with a RSV infection rate higher than 80%, was screened and reared in the lab on rice seedlings grown in 1 L beakers at 25 °C with a photoperiod of 16 h /8 h (light/dark).

The RBSDV-infected SBPH vectors were prepared as described previously [20]. Non-viruliferous SBPHs were fed with rice black streaked dwarf diseased plants for three days, and then transferred to healthy rice seedlings for two weeks to pass the latent period. The SBPHs were subsequently collected for RBSDV detection. RSV-free and RSV-viruliferous SBPHs in 2nd to 4th instar were mixed and used for HiPV detection and duplex RT-PCR assay.

### Crude extract preparation from a single SBPH for reverse transcription

The SBPHs reared on rice seedlings were collected and frozen in −20 °C for 5 min. A single SBPH was placed in a 0.2 mL centrifuge tube, washed with 100 μL sterile H_2_O, and ground with sterile wet toothpicks in 30 μL sterile H_2_O. After centrifugation at 12,000 g for 1 min, the supernatant from individual SBPH was immediately transferred to new 200 μL centrifuge tube and used for reverse transcription.

### RT-PCR

The crude RNA extract was used as template for simplified RT-PCR. The cDNA was synthesized using M-MuLV 1st strand cDNA synthesis kit (Sangon Biotech, P.R. China) according to the manufacturer’s protocol. The procedure is as followed: 11 μL crude sample and 1 μL random primer (Random 6, 0.2 μg/μL) were mixed and incubated at 65 °C for 5 min, the mixture then was transferred onto ice for 30 s immediately. After a short centrifuge, 4 μL 5 × M-MuLV reverse transcriptase buffer, 2 μL dNTP mix (10 mM), 1 μL RNase inhibitor (20U/μL) and 1 μL M-MuLV RT (200 U/μL) were added. The tubes were incubated in 25 °C for 10 min, 42 °C for 1 h, and 70 °C for 10 min. The resulting cDNA could be stored in −20 °C or applied to virus PCR detection directly.

Specific virus primers were used for PCR amplification to detect viruses in a single SBPH (Table 1). PCRs were performed with a final reaction volume of 20 μL, containing 5 μL cDNA, 10 μL 2 × Taq Master Mix (Vazyme Biotech, P.R. China), 0.5 μL each of the primers. The initial denaturation (95 °C, 5 min) was followed by 40 cycles of 95 °C for 30 s, 58 °C for 30 s, 72 °C for 1 min, and a final extension step at 72 °C for 10 min. A plasmid containing the RSV CP gene was used as PCR positive control for detection of RSV and a plasmid with the RBSDV P10 gene for RBSDV. Crude extracts from non-viruliferous SBPH were used as negative controls. The PCR products were evaluated by agarose gel electrophoresis.

**Table 1:** Primers used for RT-PCR or qRT-PCR amplification of RSV, RBSDV and HiPV

Name	Sequences (5′→3′)	Accession number	Amplicon length (bp)
RSV CP-F1	ATGGGTACCAACAAGCCAGC	EF198700	936
RSV CP-R1	CTAGTCATCTGCACCTTCTG		
RBSDV P10-F	ATGGCTGACATAAGACTCGA	NC_003733	1677
RBSDV P10-R	TCATCTTGTCACTTTGTTTA		
HiPV-F	CTGGACAACATGATATTAGA	AB183472	678
HiPV-R	CTATTTCCCAGTTCCAAG		
RSV CP-F2	GCCACTCTAGCTGATTTGCA	EF198700	167
RSV CP-R2	GTGTCACCACCTTTGTCCTT		

### Dot immunobinding assay (DIBA)

Crude extracts (1 μL) were dotted individually onto a nitrocellulose membrane (0.2 μm pore size, Pall) and allowed to dry at room temperature. Nonspecific sites were blocked with blocking buffer containing 2% skim milk in PBST (137 mM NaCl, 2 mM KCl, 10 mM Na_2_HPO_4_, 2 mM KH_2_PO_4_, pH7.5, 0.05% Tween-20) at 37 °C for 30 min. The membrane was subsequently incubated in blocking buffer containing RSV- or RBSDV-specific monoclonal antibody at 1:5,000 dilution at 37 °C for 1.5 h. After wash for three times (each 5 min) with PBST, membranes were immersed in secondary antibody conjugated with HRP (Sigma-Aldrich, USA) at 1:2,000 dilution with PBST containing 2% skim milk. After another round of washes (3 times), the membranes were developed in a freshly prepared substrate solution containing 6 mg 4-chloro-1-naphtol, 2 mL ethanol, and 7 μL of 30% H_2_O_2_ in 10 mL PBS. The crude extracts of the individual SBPHs that developed well-defined dots and those with no dots on the membranes were kept in −20 °C, and used as positive and negative controls for the DIBA assay.

### Comparison of sensitivity of virus detection with and without RNA isolation

To compare the detection sensitivities of the simplified RT-PCR using crude extract and the traditional RT-PCR using purified RNA, RSV RNA in individual SBPHs was prepared by these two methods and detected using RSV CP primers. Individual SBPHs were ground as described above. An aliquot of 11 μL supernatant was immediately transferred to a new tube for centrifugation and then the crude extract was used for reverse transcription. Another aliquot of 11 μL supernatant was used for Trizol RNA isolation. The reverse transcribed products were evaluated by PCR and qRT-PCR.

As SBPHs cannot be completely ground by toothpick, the tissue in the same volume of the supernatant (11 μL) may not be equal. In order to ensure that the two methods processed the same amount of tissue sample, the RSV-viruliferous SBPHs were treated in another way. Every 10 SBPHs at the third instar stage were collected in a 1.5 mL centrifuge tube and ground in liquid nitrogen using a pestle. Then 100 μL RNase-free ddH_2_O were added. After thorough vortexing, 11 μL suspensions was transferred to a new tube as crude extract and another 11 μL suspension was used for Trizol RNA isolation.

### Trizol RNA isolation

Isolation of total RNAs with Trizol (Invitrogen, USA) was operated according to the manufacturer’s protocol with some modifications. An aliquot of 11 μL of supernatant was transferred to a new 1.5 mL tube. Subsequently 120 μL Trizol and 26 μL chloroform were added and mixed well. After centrifugation of the tube at 12,000 g for 15 min at 4 °C, the upper aqueous phase was immediately transferred into a new 1.5 mL Eppendorf tube. An equal volume of isopropanol was added to the aqueous layer, vortexed and placed in room temperature for 10 min. After centrifugation at 12,000 g for 10 min at 4 °C, the supernatant was removed and the RNA pellet was rinsed with 200 μL 75% ethanol twice. The RNA was dried and resuspended in 11 μL RNase-free ddH_2_O. The total RNA was used for reverse transcription or stored at −70 °C.

### qRT-PCR

qRT-PCR was performed with an IQ5 Real-Time PCR System (Bio-Rad, USA) in a final reaction volume of 20 μL, containing 8 μL ddH_2_O, 0.5 μL of each primer, 1 μL of cDNA template and 10 μL of 2 × SYBR@premix Ex Taq II (Tli Rnase H plus) (TaKaRa, including SYBR Green I, TaKaRa Ex Taq HS, dNTP mixture, Mg^2+^ and Tli RnaseH). The PCR was run as described previously [20].

## Results

### The simplified RT-PCR method detected RSV in single SBPHs

**Fig. 1:**
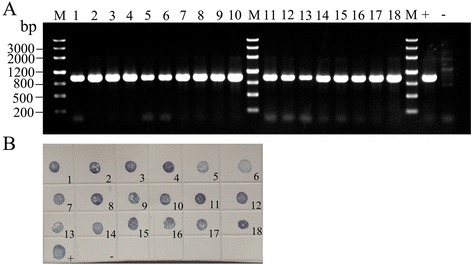
RSV detection in individual SBPHs. **a** PCR results of the RSV detection using the simplified RT-PCR; (**b**) RSV detection by DIBA assay

The simplified RT-PCR was used to amplify the RSV CP gene in the RSV-viruliferous SBPH. The designed primers specifically amplified a fragment of 936 bp, which is consistent with the expected size (Fig. 1a). The PCR products were further confirmed to be the RSV CP gene (Additional file 1) by sequencing. The results indicated that RSV was successfully detected by the simplified RT-PCR. To confirm the results, the crude extracts from individual SBPHs were also processed by DIBA assay using RSV monoclonal antibody. As shown in Fig. 1b, the results of DIBA assay are consistent with those of RT-PCR.

### The simplified RT-PCR method detected RBSDV in single SBPHs

**Fig. 2:**
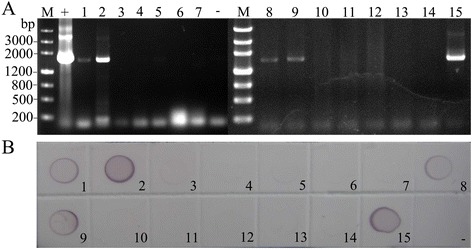
RBSDV detection in individual SBPHs. **a** PCR results of the RBSDV detection using the simplified RT-PCR; (**b**) Detection of RBSDV by DIBA assay

In addition to detection of RSV in individual SBPHs, we also detected RBSDV using the simplified RT-PCR method without RNA isolation. The specific fragments with expected size of 1,677 bp were obtained in positive samples and confirmed to be the RBSDV P10 gene by sequencing (Additional file 1); no bands were amplified in the non-viruliferous SBPH samples (Fig. 2a), indicating that RT-PCR using crude extract successfully detected RBSDV in a single SBPH. The results were further validated by DIBA using RBSDV monoclonal antibody (Fig. 2b). The data confirmed that this simplified RT-PCR method was reliable for detecting RNA virus in SBPH.

### HiPV was detected in single SBPHs

**Fig. 3:**
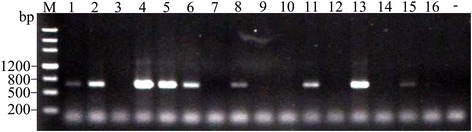
Detection of HiPV in single SBPHs using the simplified RT-PCR

HiPV was found in SBPH in early 1990s [6, 21] and its existence was confirmed by high-throughput sequencing [17]. We used the RT-PCR method without RNA isolation to analyze whether HiPV existed in our laboratory-reared SBPH. The results showed that the specific bands with expected size of 678 bp were successfully amplified in HiPV-infected SBPHs (Fig. 3). Sequencing results revealed that the PCR products were specific to the corresponding virus (Additional file 1), which validated the specificity and reliability of the assay.

### Duplex RT-PCR simultaneously detected two RNA viruses in single SBPHs

**Fig. 4:**
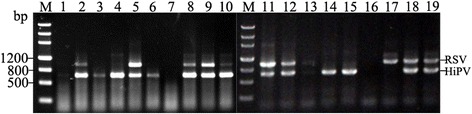
Simultaneous detection of HiPV and RSV by duplex RT-PCR without RNA isolation

To test whether the cDNA prepared from crude extract could be used for simultaneous detection of multiple RNA viruses, duplex RT-PCRs were performed to amplify RSV and HiPV in a single SBPH. The primer pairs for the two viruses were mixed and used for virus detection. A 678 bp PCR product corresponding to RSV or a 936 bp corresponding to HiPV was detected in SBPHs infected only by RSV or HiPV; the samples infected with both viruses detected both fragments of 678 bp and 936 bp (Fig. 4).

### Analytical sensitivity of the simplified RT-PCR

**Fig. 5:**
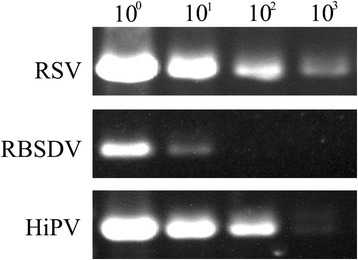
Detection of RSV, RBSDV and HiPV by simplified RT-PCR in a 10-fold serial dilution

To evaluate the relative sensitivity of this method, a series of 10-fold dilutions of the cDNA obtained from crude extracts were used for PCR analysis. Consistent with the different accumulation in individual SBPHs (shown in Figs. 1, 2 and 3), the detection sensitivity varies in different virus species. RSV and HiPV could be detected up to 10^3^ fold dilution, although the HiPV specific band was not distinct at 10^3^ fold dilution. RBSDV could be detected by the presence of an expected PCR product when the crude extracts were diluted 10 fold (Fig. 5).

### Comparison of the sensitivity of RT-PCR with or without the RNA isolation step

**Fig. 6:**
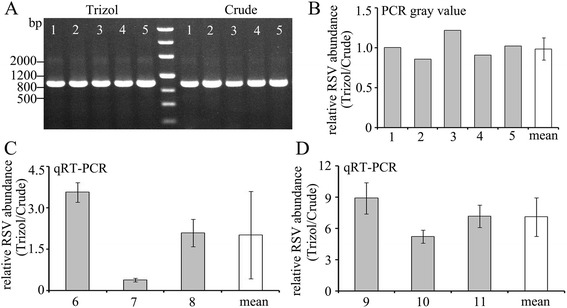
Comparison of the sensitivity of simplified RT-PCR (Crude) and traditional RT-PCR (Trizol). **a** PCR products amplified from samples prepared by the two methods. **b** Relative RSV abundance evaluated by gray value of PCR products. **c** Relative RSV abundance evaluated by qRT-PCR in the samples ground by toothpick. **d** Relative RSV abundance evaluated by qRT-PCR in the samples ground in nitrogen liquid

To compare the detection sensitivity of the simplified RT-PCR and traditional RT-PCR, single RSV-viruliferous SBPHs were ground and RSV in the same volume of the crude suspension was detected by these two methods. The RSV CP gene could be detected in all the five independent samples by both methods (Fig. 6a). RSV abundance was measured by scanning the gray value of the PCR bands. The ratio of the gray values of PCR product amplified from Trizol extracted RNA and crude samples (Trizol/crude) was close to 1.0 (Fig. 6b), indicating that there are no obviously differences in the detection sensitivities between the simplified RT-PCR and traditional RT-PCR.

To more accurately evaluate the detection sensitivities of these two detection methods, we used qRT-PCR to compare the RSV abundance in the samples prepared by the two methods. The RSV abundance in the samples was evaluated by the CT value of qRT-PCR. The virus abundance in the crude samples was set as the reference, and the relative virus abundance in the samples prepared by traditional Trizol extraction (Trizol/Crude) was calculated by 2^△CT(Crude-Trizol)^. The results showed that the viral RNA abundance in samples #6 and #8 extracted using Trizol were higher than those in crude extracts, while sample #7 showed the opposite result (Fig. 6c). We speculated the reason was that the SBPH was not crushed completely by toothpick and thus the tissues could not be divided evenly.

In order to avoid this problem, we further ground 10 SBPHs in nitrogen liquid and distributed equal amount of tissues for further processing by the two different methods; samples #9, #10, and #11 represent this preparation. The qRT-PCR results show that the relative RSV abundance in the samples using Trizol isolation contains an average RSV level seven fold over those prepared using the simplified method (Fig. 6d).

Monitoring the ratio of viruliferous SBPH in the field insect population is important for viral disease forecasting and control. To test whether the simplified RT-PCR could be used for field insect samples, we collected field insect samples from Kaifeng, Henan Province, China for RBSDV detection. Ninety six SBPHs were randomly selected to detect RBSDV using the traditional RT-PCR method and the simplified RT-PCR established in this study. A single SBPH was ground in 30 μL sterile H_2_O. After centrifugation, 11 μL supernatant was used for detection by the simplified RT-PCR and the rest of the sample by traditional RT-PCR. The results obtained by the two methods are consistent; both methods showed that three samples were RBSDV positive (Additional file 2: Figure S1), suggesting that the simplified RT-PCR is reliable for detecting virus in field insect samples.

## Discussion

Plants are infected by a wide range of viruses that cause economic losses and pose threats to certain agricultural industries. Hemipteran insects act as the main vectors for the plant viruses, transmitting as much as 55% of the described plant viruses. Planthoppers in Hemiptera mainly transmit RNA viruses: among the 18 viruses they transmit, 14 are RNA viruses [22]. Analysis of virus infection in the insect vector is important for preventing the potential disease threat. In this study, we developed a simplified RT-PCR method without RNA isolation for RNA virus detection in a single SBPH. The virus RNA preparation can be completed in approximately 2 min and requires no pre-treatments for RNA purification. This simplified method may be expanded for detection of RNA viruses in other insects in Hemiptera with similar insect size, such as aphids, leafhoppers, brown planthopper and whitebacked planthopper.

The traditional RT-PCR for RNA virus detection contains a laborious RNA isolation process [18]. There are several methods to isolate RNA from insects, including using commercial SV total RNA isolation system (Promega) [23], Trizol reagent (Invitrogen) [24], and the RNAiso Reagent (TaKaRa) [15]. The RNA samples isolated by these methods are of high quality with limited contamination and can produce well-defined amplification products in RT-PCR [25]. However, it is expensive to use these commercial kits. Besides, it is time-consuming and inconvenient to extract RNA from a single SBPH because planthoppers are of small size. Our method offers an effective way to detect viruses in a large number of planthopper samples by eliminating the expensive and laborious RNA isolation process.

The quality of RNA is considered as one of the important factors that affect the accuracy of RT-PCR. RNA samples with their A260/A280 ratios falling between 1.8-2.0 are considered to be of good quality and selected for RT-PCR and subsequent analysis [26]. The A260/A280 ratios of crude extracts of SBPH in our assay are below 1.8 (data not show) and the crude extracts are presumed to contain contaminating components, such as DNA, protein and lipid, which might inhibit RT-PCR reactions. However, the simplified RT-PCR successfully detected the RSV, RBSDV and HiPV in SBPH (Figs. 1, 2 and 3). Moreover, the cDNA prepared by the described protocol can be used for duplex RT-PCR and qRT-PCR for virus detection (Figs. 4, Fig. 6c and d). These results suggest that the contaminants in crude extracts do not affect the detection results, which is in agreement with previous studies showing that viruses could be detected specifically in plant samples by RT-PCR using unpurified RNA [27]. One possible explanation is that the contaminants in planthopper extracts that can inhibit RT-PCR reactions are present at very low concentrations, not high enough to cause problems in RT-PCR.

On the other hand, we found that the reverse transcription method affects the result of the simplified RT-PCR. When we first incubated the crude extract at 65 °C with random primer (Random 6) and then proceeded to reverse transcription as described in the Methods, the RNA virus was successfully detected (Figs. 1, 2 and 3). However, when the cDNA was obtained by using the primeScript™ RT Master Mix (Takara, contains every components except the RNA template for reverse transcription) without heat treatment, the RNA virus could not be detected by the simplified RT-PCR. We hypothesize that virus particles in SBPH may require heat disruption at 65 °C to release RNA for cDNA synthesis. Alternatively, contaminating inhibitors may be inactivated by treatment at 65 °C.

The sensitivity of the detection method is another important factor for virus detection. Our results show that the simplified RT-PCR method bears the sensitivity sufficient to reliably detect RSV, RBSDV and HiPV in a single SBPH (Figs. 1, 2 and 3). Both RSV and HiPV were detected when cDNA samples were diluted up to 10^3^ fold (Fig. 5). These results suggest that the simplified method can be practically applied to virus detection using a single SBPH. The relative abundance of RSV analyzed by qRT-PCR using Trizol-isolated RNA was 7 fold higher than that using RNA in crude extract (Fig. 6d). One explanation is that the Trizol reagent disrupts insect tissues more thoroughly to release RNA from tissues whereas some amounts of RNA may be lost together with tissues during centrifugation in the simplified method.

## Conclusions

A simplified RT-PCR method was developed for detection of RNA virus in a single SBPH by preparing virus RNA in an easy and fast procedure. Our results demonstrated that crude extracts of SBPHs could be used as the template for RT-PCR. The viral RNA prepared by this method was also suitable for duplex RT-PCR and qRT-PCR detection. This protocol reduces the use of costly reagents, shortens the sample processing time, and improves the efficiency of virus detection. As the method is highly simplified and of sufficient sensitivity, it provides a useful tool to the investigation of epidemics of viral diseases in the early stage by enabling easy detection of viruses within a single insect vector.

## Additional file

Additional file 1: Sequencing results of the detected viruses. (DOCX 14 kb)
Additional file 2: Figure S1. Detected RBSDV in individual field-collected SBPH using the simplified RT-PCR and traditional RT-PCR. (A) PCR products of 96 field-collected SBPH detected by simplified RT-PCR. (B) PCR products of SBPHs detected by traditional RT-PCR. (JPG 1439 kb)

## Abbreviations

DIBA: Dot immunobinding assay
HiPV: Himetobi P virus
qRT-PCR: Quantitative real time PCR
RBSDV: Rice black streaked dwarf virus
RSV: Rice stripe virus
RT-PCR: Reverse transcription polymerase chain reaction
SBPH: Small brown planthopper
