# Supplementary material for: Development of a simplified RT-PCR without RNA isolation for rapid detection of RNA viruses in a single small brown planthopper (Laodelphax striatellus Fallén)
Source: Virol J. 2017 May 3;14:90. doi: 10.1186/s12985-017-0732-6 (PMC5415734; doi:10.1186/s12985-017-0732-6)
Supplement: Additional file 1: — Sequencing results of the detected viruses. (DOCX 14 kb) [file 12985_2017_732_MOESM1_ESM.docx]

Additional file 1

The sequencing results of RSV PCR product are consistent with the RSV *CP* gene and the detailed sequence information is shown below:

ATGGGTACCAACAAGCCAGCCACTCTAGCTGATTTGCAGAAGGCAATCAATGACATCTCCAAAGATGCGTTGTCTTACCTGACTGCTCATAAAGCTGATGTTGTGACCTTTGCTGGTCAGATAGAGTATGCAGGCTATGATGCTGCAACTCTGATTGGCATATTGAAGGACAAAGGTGGTGACACACTGGCCAAGGATATGACTATGTGCATCACCATGAGATATGTGAGAGGCACTGGCTTTGTGAGAGATGTCACTAAGAAAGTGAAAGTGGCGGCTGGAAGCACAGAGGCTTCGACCTTGGTGTCGAGGTATGGGATAGTGTCCTCGGTGGGGACAAATGCCAATGCTATCACACTTGGAAGGCTGGCTCAGCTATTCCCAAATGTCTCACATGAAGTTGTGAGACAAATTTCTGGTGTTAAGATGGCTGTGGACTCCTCTGACCTGGGACTAACAGGATGTGATAACTTACTGTGGGACTATGTTCCACAATATATTAAACTGGAGAGTGAAACAGCTCCTTACTGCACAACTCACTCCCTAAGTCACATTTTGTTTGTTGTGCACATCATTCACTCCTTCCAAATAACCAAAAAGACCATGCCAGAGGGTAAGAAGAAGGAGCGTGGTCTGACAAAAGACATAGACATGATGAAGTACACAACTGGTCTCCTGGTCATCACATGCAGGTCAAAGAACCTGGCTGACAAGAAGAAGGAAGATGGCAGAAAGAAGGTCTTAGATGAATTCATCACCAATGGGAAAGTGAAGACCACAATCTTCGATGCGCTGGCTGGTATGTCTGTCAATACGATCAGCACTTATGGGAATCAGACAAGGCTGTACTTGGCTCAACAGAGCAAACTGATGAAGATCCTTGCTGAGAACACTTCAAAGACAGCATCTGAAGTCAGCGGGTTGGTGAAGGAGTTCTTCGAGGACGAGGCAGAAGGTGCAGATGACTAG

The sequencing results of RBSDV PCR product are consistent with the RBSDV *P10* gene and the detailed sequence information is shown below:

ATGGCTGACATAAGACTCGACATAGCGCCCGATCTTATCCATAATGGTGTACCCCAGAGACTTTCCGATACAATAATTTTAAACAACCGACCAACAATCACTCTGTTATCTCATTTCAACAATCTATTTCATGAATTAAACATTGTCAAAGCGCCCCACGTTGCATCTTCCCAAACTACCGTTAATTTGTACATTCGCAAACATTTGTTGACCCGACTTCATGATAGACTACAAACCGTAGAAACTAGCACTTTACCCAACATCACTCAACTTAAAGACCACATTCGCAGTTTCTTTCAAAATGAACACCAACCCATTTTTCAGACCCTAACGAACAACGACCTAAGCGAAGAATTTGTAGGTGTGACTACTTTTGGACTAAGCTTATTTGCTACCTCCAAACTTGATGCTGAACAAATAGAACGTGTGCAAATTGAGACCTTAACTGAAGGAAACATTACGTTGAAGCCCTTTTCCGCTGATGGTTTAGAAGTCATTCTCGATGATAGTTATATTGGTATAGTTGGCAAAATTCCAGGTTTAGAAGTTCATAAATTGTTAGATAAATGTTGTCGTGAAGTTCCTGCTCAAATGGGAATACTTACTGATGAAGTTAAACTTTTGATGCGTACTGGTAAATTAAGAATTGATGGTGGTTACGATTTCAATTGTCCTGCAAGCACTACAGATGTTACACACTACGGTGGTTATGACCAATTTTCGCGTCAAATGTTCGAACGTTTGAATCTTTTTTATAATATTAGTCTTAGCATAATTCCCGTTTCAGCTTTAAAAACAGTTCATTTATTCGAAAAAGAATTAAGTGTTTTGGATGCAGACAAATCTTTACTCGAACAGACTTGGAGCGCAGTAGCGTCATTTGTTGAAACCTGGCAAGTTAAATCTAAAGTTAAAGCTGATGATCCTGATGAATACGAACTGACCAGTTTGTCCACTTTGCGTACTAATTATGATGGTACTTCTACTTCTAGCCCTTTTACAGATAAGAAATTCATTGACTGGTATATTAAGACTTTTTCTAAGACTGAAAAAGGATCGTCTTTACGTCGAAATGAATTAGAAGAGAAAAGTGCCACTAATACTTCAACTACAGTAAAGAAGGTTAAAATTCATTTCTCTGTTCAATATTTTGACGAATTTAAAGTTAATGGACACGAAAAAAGCATTGTAGTTCAAACTCATAAAGGTGAAATGACACTAGATTATTATCGTAAAATTGGCGAAGTATTAAGTGCGATTTGGAAACGTGGTAAATCTTTGGCTGTACCTTGTTTTGATTACATTAAACTTGGCGTTGAAAAAGCATTTCATTTGGCACCCGTAATTATGAAGAAGTATAACTTGACGATCGACGATATTATCAACTTCATTGATAAAGGACCTTCCTATTTGGCTAAGTTGGACAAAATTGATGATTGGTCTCTGATTTCAAAGCTTATTATTACCAGTGTTTTACCTAACATTATTCAAGCTGTTTATAAAACCGATCCAAGTAATAATGTTATGAACTCAGTAATTATCAGTAGAGCGAACAACTTGTTGAAATCTGATAGGGACAGGCTATTAAAGAAGGCACTTTCCGCCAACGTTTCTTCCTCCAATACTTCTAGTCATGAGCATACACAGAAGATAGTATTAAACAAAGTGACAAGATGA

The sequencing results of HiPV PCR product are consistent with the HiPV genome sequence and the detailed information is shown below:

CTGGACAACATGATATTAGATCTGATTATTTAGAAGATAAGATAGAAATTAAAGATATTACTGGAATTCCGTCTAATGTATCCCTAAATACTGAAAAATCCCTATCCTGTGTTGGAGAGTCATTTGGAAATTTTCGTGATTTTATTAAGCGGTTTGGTTGGCATAAAAGCCAATCGGTCGCTTCTTCAAACACGAAGATCCTGAGTGGCATTCCAATTGTTAATTATACATCAAGTATTTCTGGCACAGGTCTCACGCTGACAGCTGATGGAGGCTCTACACCACTAACAATGGTGAGTAGCATGTATGCTTTCTTTCGCGGAGGTTTTCGCGCGAAAGTATACATCCATGATCTGCCTGCGGGAGAAATGGTCCAGGGTGCTTTGATTGATAATTCACAAACCACAAATGTTCCCCAACCCCTTGCCCTACAATCCCTTCAATATGAATTGTCTGATAAGAGACTTTATGAGTTCTCCTGGCCTTACTATTGCCCCACATATTTAACGACCTATCCTTCTGGGTCGCTAAATTATATTTCCGATCTAGTGAATCCCACAACGTATGCACGCATCACAACCATCAGTGAGTACGCTACAGCTTATGCTATGGCTGCTGCTGATGATTTTGATTGCGGGTTTTACTTGGGGGCTCCATTATCTTGGAACTGGGAAATAG
