# Supplementary figures and images for: Development of a simplified RT-PCR without RNA isolation for rapid detection of RNA viruses in a single small brown planthopper (Laodelphax striatellus Fallén)
Source: Virol J. 2017 May 3;14:90. doi: 10.1186/s12985-017-0732-6 (PMC5415734; doi:10.1186/s12985-017-0732-6)

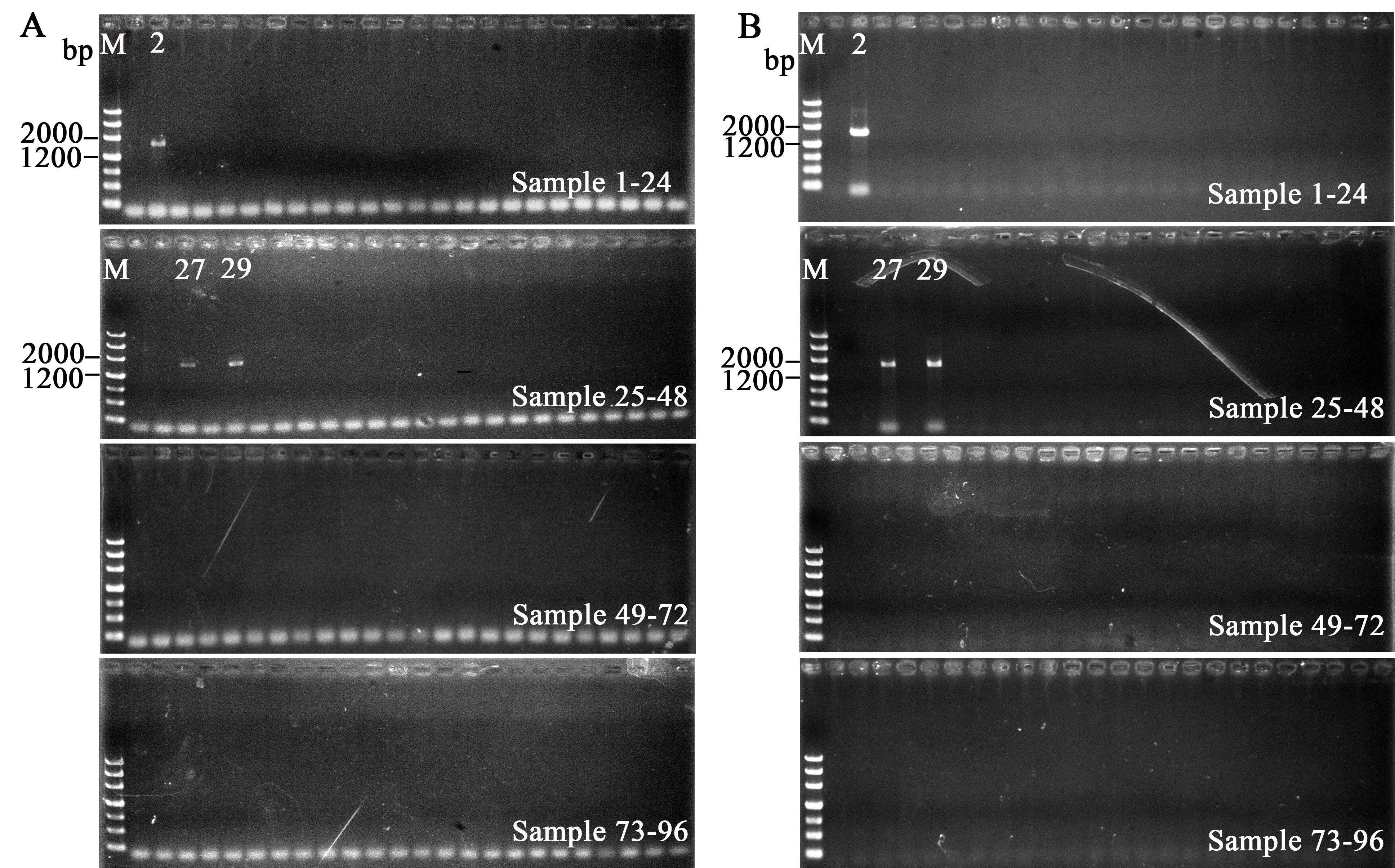

Supplement: Additional file 2: Figure S1. — Detected RBSDV in individual field-collected SBPH using the simplified RT-PCR and traditional RT-PCR. (A) PCR products of 96 field-collected SBPH detected by simplified RT-PCR. (B) PCR products of SBPHs detected by traditional RT-PCR. (JPG 1439 kb) [file 12985_2017_732_MOESM2_ESM.jpg]
